# Supplementation of a grape seed and grape marc meal extract decreases activities of the oxidative stress-responsive transcription factors NF-κB and Nrf2 in the duodenal mucosa of pigs

**DOI:** 10.1186/1751-0147-55-18

**Published:** 2013-03-02

**Authors:** Denise K Gessner, Anja Fiesel, Erika Most, Jennifer Dinges, Gaiping Wen, Robert Ringseis, Klaus Eder

**Affiliations:** 1 Institute of Animal Nutrition and Nutrition Physiology, Justus-Liebig-Universität Gießen, Heinrich-Buff-Ring 26-32, 35392, Gießen, Germany

**Keywords:** NF-κB, Nrf2, Polyphenol, Pig, Intestine

## Abstract

**Background:**

In pigs, enteric infections and the development of gut disorders such as diarrhoea are commonly observed, particularly after weaning. The present study investigated the hypothesis that feeding a grape seed and grape marc extract (GSGME) as a dietary supplement has the potential to suppress the inflammatory process in the small intestine of pigs by modulating the activities of NF-κB and Nrf2 due to its high content of flavonoids.

**Methods:**

Twenty-four crossbred, 6 weeks old pigs were randomly assigned to 2 groups of 12 animals each and fed nutritionally adequate diets without or with 1% GSGME for 4 weeks.

**Results:**

Pigs administered GSGME had a lower transactivation of NF-κB and Nrf2 and a lower expression of various target genes of these transcription factors in the duodenal mucosa than control pigs (*P* < 0.05). Concentrations of α-tocopherol and thiobarbituric acid reactive substances (TBARS) in liver and plasma and total antioxidant capacity of plasma and relative mRNA abundances of NF-κB and Nrf2 target genes in the liver did not differ between the two groups. However, the ratio of villus height:crypt depth and the gain:feed ratio was higher in the pigs fed GSGME than in control pigs (*P* < 0.05).

**Conclusions:**

This study shows that dietary supplementation of a polyphenol rich GSGME suppresses the activity of NF-κB in the duodenal mucosa of pigs and thus might provide a useful dietary strategy to inhibit inflammation in the gut frequently occurring in pigs. Feeding GSGME did not influence vitamin E status and the antioxidant system of the pigs but improved the gain:feed ratio. In overall, the study suggests that polyphenol-rich plant extracts such GSGME could be useful feed supplements in pig nutrition, in order to maintain animal health and improve performance.

## Background

In pigs and other monogastric animals, the weaning phase is a critical and stressful period which is commonly accompanied by an increased susceptibility to enteric infections and the development of gut disorders such as diarrhoea [1]. This increased susceptibility has been explained by an inflammatory process induced in the intestine during weaning [1,2]. The inflammatory process is mainly triggered by the transcription factor nuclear factor kappa B (NF-κB) [3,4]. After activation by various inducers including cytokines, reactive oxygen species (ROS) and bacterial lipopolysaccharides, NF-κB translocates from the cytoplasm to the nucleus and activates the expression of a wide variety of genes encoding pro-inflammatory proteins such as cytokines, chemokines, adhesion molecules or enzymes involved in the inflammatory process [5-7]. These inflammatory mediators as well as ROS, produced in the course of the inflammatory process, contribute to the disruption of the epithelial barrier and favour attraction and migration of other immune cells which enhance the inflammation of the intestine in pigs [8]. As a counterpart of NF-κB, nuclear factor-erythroid 2-related factor-2 (Nrf2) – a redox sensitive transcription factor - plays a protective role in inflammation and responds to pro-inflammatory stimuli and therefore rescues cells from inflammatory injuries [9,10]. Nrf2 modulates cellular defense against oxidative and electrophilic insults by rapid induction of antioxidative and phase-II detoxifying enzymes and related stress-response proteins [11]. Under normal conditions, Nrf2 is retained in the cytoplasm by interaction with Kelch-like ECH-associated protein 1 (Keap1). Oxidative stress causes dissociation of Keap1 from Nrf2 which allows Nrf2 to translocate into the nucleus, activating antioxidant and cytoprotective genes by binding to antioxidant response elements (ARE) in the promoter regions of its target genes [12].

Since inflammation of the intestine in piglets not only impairs function and integrity of the gut but also affects growth performance, dietary strategies to inhibit the inflammatory process in the small intestine are in great demand. There is evidence from numerous studies in humans and animals that dietary polyphenols, especially some representatives from the group of flavonoids are able to attenuate inflammation through modulation of the activities of NF-κB and Nrf2 [10,13,14]. However, the potential anti-inflammatory effects of polyphenols have been scarcely investigated so far in farm animals.

By-products of wine/grape juice processing provide an abundant and inexpensive source of flavonoid compounds. In the present study, we investigated the hypothesis that feeding a grape seed and grape marc extract (GSGME) as a dietary supplement has the potential to suppress the inflammatory process in duodenum of piglets by modulating the activities of NF-κB and Nrf2 due to its high content of flavonoids. In order to assess a possible effect of this feed supplement on the antioxidative status, we also determined tocopherol concentrations, antioxidative capacity and concentrations of lipid peroxidation products in plasma and liver of the pigs.

## Materials and Methods

### Animals and diets

Twenty-four (12 male, 12 female) six week old crossbred pigs (Danzucht × Pietrain) were weighed and randomly assigned to two groups of 12 pigs each. The pigs were housed individually in flat-deck pens with controlled temperature (23 ± 2°C), relative humidity (50-60%), and light from 06.00 to 19.00. They were given water *ad libitum* from a nipple drinker system during the whole experiment and fed two nutritionally adequate basal diets in phases I (<15 kg body weight) and II (>15 kg body weight), according to the German Society for Nutrition Physiology (GfE) [15] for a whole period of 28 days. Composition and nutrient concentration of the diets are shown in Table 1. The GSGME group received the same diet supplemented with 1% of a commercial feed additive consisting of grape seed and grape marc meal (Anta^Ox^ E, Dr. Eckel GmbH, Niederzissen, Germany). The composition of the GSGME (*Vitis vinifera* L.) was as follows (in g/kg): crude fibre (346), crude protein (110), crude fat (41), crude ash (29). The total polyphenol content was 8.5% according to the manufacturers' specification. To control the feed intake, unconsumed feed was weighed daily. The metabolisable energy of the diet was calculated as recommended by the GfE [15]. Concentration of crude protein in the diets was analysed according to the official German methodology of Verband Deutscher Landwirtschaftlicher Untersuchungs- und Forschungsanstalten (VDLUFA) [16]. All experimental procedures described are in strict accordance with the recommendations in the guidelines for the care and use of laboratory animals [17] and the Appendix A of European Convention for the Protection of Vertebrate Animals used for Experimental and other Scientific Purposes [18]. In Accordance with Article 4 par. 3 of the German Animal Welfare Law [19] all animals were humanely killed for scientific purpose approved by the Animal Welfare Officer of the Justus-Liebig-University, JLU No. 506_AZ.

**Table 1:** Composition of the basal experimental diets fed in phase I (body weight <15 kg) and II (body weight >15 kg)

	**Phase I**	**Phase II**
**Composition (g/kg)**		
Wheat	381.7	406.9
Barley	315	302
Soy bean meal (44% crude protein)	250	240
Soy oil	15	15
Mineral and vitamin premix*	33.5	33.4
L-Lysine	2.6	1.5
DL-methionine	1.0	0.5
L-threonine	1.2	0.7
**Concentration of nutrients**		
Metabolisable energy (MJ/kg) †	12.9	13.4
Dry matter (%) ‡	88.7	89.4
Crude protein (%) ‡	19.6	19.1
Crude fibre (%) ‡	5.3	4.9
Crude fat (%) ‡	3.4	4.4
Crude ash (%) ‡	4.8	5.1
Digestible lysine (%) ¥	1.16	1.05
Digestible methionine + cysteine (%) ¥	0.62	0.57
Digestible threonine (%) ¥	0.69	0.63
Digestible tryptophan (%) ¥	0.21	0.21

*The mineral and vitamin premix (Bergin Novamast, Bergophor, Kulmbach, Germany) provided the following per kg diet: 1.34 g lysine, 1,020 FYT phytase, 102 mg iron, 102 mg manganese, 102 mg zinc, 20.4 mg copper, 2.21 mg iodine, 0.44 mg selenium, 13,400 IE vitamin A, 2,244 IE vitamin D_3_, 102 mg vitamin E (α-tocopherol acetate), 2.55 mg vitamin K, 2.55 mg vitamin B_1_, 6.8 mg vitamin B_2_, 5.1 mg vitamin B_6_, 34 μg vitamin B12, 34 mg nicotinic acid, 17 mg Ca-D-pantothenic acid, 1 mg folic acid, 136 μg biotin, 340 g choline chloride.

† Calculated according to recommendations of German Society for Nutrition Physiology.

‡ Analysed (mean values of three analyses per diet).

¥ calculated using tabular values from AMINODat^®^ 4.0 (Evonik Industries AG, Essen, Germany).

### Sample collection

After 4 weeks, body weights of all pigs were recorded and pigs were anaesthesised and exsanguinated for sample collection. Blood samples were collected into heparinised polyethylene tubes. Plasma was obtained by centrifugation of the blood samples (1100 g, 10 min, 4°C) and stored at −20°C. An accurately defined piece of the duodenum was removed and rapidly excised, flushed with a 0.9% sodium chloride solution and cut open longitudinally to scrape off the mucosa with a glass slide. Tissue samples of liver and mucosa were snap-frozen in liquid nitrogen and stored at −80°C pending analysis.

### Trolox equivalent antioxidant capacity (TEAC), concentrations of thiobarbituric acid-reactive substances (TBARS) and α-tocopherol

Trolox equivalent antioxidant capacity was determined in liver and plasma by high performance liquid chromatography (HPLC) with fluorescence detection according to Balz et al. [20]. In brief, samples of plasma (200 μl) or liver (100 mg) were mixed with 2 ml of 1% pyrogallol (in ethanol) solution and saponified with 300 μl saturated sodium hydroxide solution. This mixture was heated for 30 min at 70°C. Tocopherols were extracted with n-hexane, separated isocratically on a C-18-reversed phase column [Luna C18 (2), Phenomenex, Aschaffenburg, Germany], using methanol as mobile phase and detected by fluorescence (excitation wavelength: 290 nm, emission wavelength: 325 nm).

TBARS were measured in plasma and liver using a modified version of the method of Sidwell et al. [21]. Sample aliquots were mixed with thiobarbituric acid reagent [thiobarbituric acid (8 g/l)/perchloric acid (70 g/l), 2:1 (v/v)] and heated for 60 min at 95°C. TBARS were extracted with n-butanol and absorption was measured at 532 nm. Concentrations were calculated via a standard curve with 1,1,3,3-tetraethoxypropane and in case of plasma, related to the concentrations of cholesterol plus triglycerides. The concentrations of cholesterol and triglycerides were determined using the enzymatic kits Fluitest^®^ CHOL (Cat.-No. 4241) and Fluitest^®^ TG (Cat.-No. 5741, Analyticon, Lichtenfels, Germany).

The Trolox equivalent antioxidant capacity of plasma was determined following the protocol of Re et al. (1999). Oxidation of 2,29-azinobis-(3-ethylbenzothiazoline-6-sulfonic acid) (ABTS) with potassium persulfate generates the blue/green radical mono cation ABTS^·+^ which is reduced in the presence of hydrogen-donating antioxidants including hydrophilic and lipophilic antioxidants. Antioxidants contained in the sample reduce ABTS^·+^ to ABTS and therefore cause decoulorisation proportional to their concentration. The absorbance was measured in a microplate-reader (Infinite^®^ M200, Tecan, Germany) at a wavelength of 600 nm and antioxidant capacity of the plasma was calculated against trolox as a standard. TEAC values expressed the mmols of trolox having the antioxidant capacity corresponding to 1.0 mmol of the test substance [22].

### Total RNA isolation, cDNA synthesis and quantitative real-time polymerase chain reaction (qPCR) analysis

Total RNA isolation by TRIzol reagent (Invitrogen, Karlsruhe, Germany) and cDNA synthesis from liver tissue and mucosa were carried out as described recently in detail [23]. Concentration and purity of total RNA were estimated from the optical density at 260 and 280 nm, respectively. RNA samples were analysed by formaldehyde-agarose gel electrophoresis, and integrity was confirmed by visualisation of 18S and 28S rRNA bands. The mRNA expression of genes was measured by real-time detection PCR using 2 μl cDNA and 18 μl of a mixture composed of 10 μl KAPA SYBR FAST qPCR Universal Mastermix (Peqlab, Erlangen, Germany), 0.4 μl each of 10 mM forward and reverse primers and 7.2 μl DNase/RNase free water in 0.1 ml tubes (Ltf Labortechnik, Wasserburg, Germany).

Gene-specific primer pairs obtained from Eurofins MWG Operon were designed using Primer3 and BLAST with anneling temperatures of about 60°C and mainly intron spanning (Table 2). qPCR was performed in a Rotor-Gene 2000 system (Corbett Research, Mortlake, NSW, Australia). To verify the presence of a single PCR product, a melting curve analysis was performed from 50°C to 95°C. To confirm the expected size of the amplificated single product, a 1.5% agarose gel electrophoresis, stained with GelRedTM nucleic acid gel stain (Biotium, Hayward, CA, USA), was used. C_t_ values of each amplification curve were obtained using Rotorgene Software 5.0 (Corbett Research, Australia). Expression values of the genes investigated were normalised using the GeNorm normalisation factor according to Vandesompele et al. (2002). To calculate the normalisation factor, all C_t_ values were transformed into relative quantification data using the 2^-ΔCt^ equation [24]. The highest relative quantities for each gene were set to 1. From these values, the normalisation factor was calculated as the geometric mean of expression data of the three most stable out of six tested potential reference genes in liver and mucosa (Table 3).

**Table 2:** Characteristics of gene-specific primers

**Gene**	**Forward primer (from 5**^**′**^** to 3**^**′**^**)**	**PCR product size (bp)**	**NCBI GenBank**
	**Reverse primer (from 5**^**′**^**to 3**^**′**^**)**		
*Reference genes*			
ATP5G1	CAGTCACCTTGAGCCGGGCGA	94	NM_001025218.1
	TAGCGCCCCGGTGGTTTGC		
ACTB	GACATCCGCAAGCACCTCTA	205	NM_001167795
	ACATCTGCTGGAAGGTGGAC		
GAPDH	AGGGGCTCTCCAGAACATCATCC	446	AF017079.1
	TCGCGTGCTCTTGCTGGGGTTGG		
GPI	CACGAGCACCGCTCTGACCT	365	NM_214330.1
	CCACTCCGGACACGCTTGCA		
RPS9	GTCGCAAGACTTATGTGACC	327	CAA23101
	AGCTTAAAGACCTGGGTCTG		
SDHA	CTACGCCCCCGTCGCAAAGG	380	DQ402993
	AGTTTGCCCCCAGGCGGTTG		
*Target genes*			
CCL2	GGTCCTTGCCCAGCCAGATGC	170	NM_214214.1
	CTGCACAGATCTCCTTGCCCGC		
CYP1A1	CTGCCATCTTCTGCCTTGTA	314	NM_214412.1
	GCTCTGGCCATTAGAGATCA		
GPX1	CTTCGAGAAGTTCCTGGTGG	232	NM_214201.1
	CCTGGACATCAGGTGTTCCT		
HMOX1	AGCTGTTTCTGAGCCTCCAA	130	NM_001004027.1
	CAAGACGGAAACACGAGACA		
HP	GTTCGCTATCACTGCCAAAC	108	NM_214000.1
	CAGTTTCTCTCCAGTGACCT		
ICAM1	CGGTGGCAGCCGTGGCTATC	208	NM_213816.1
	TTGATGCAGCCCCGCTCGTC		
IL1B	GTTCTCTGAGAAATGGGAGC	143	NM_214055.1
	CTGGTCATCATCACAGAAGG		
IL8	ACTTCCAAACTGGCTGTTGC	120	NM_213867.1
	GGAATGCGTATTTATGCACTGG		
IL6	AGCAAGGAGGTACTGGCAGA	257	NM_001252429.1
	GTGGTGGCTTTGTCTGGATT		
NQO1	CCAGCAGCCCGGCCAATCTG	160	NM_001159613.1
	AGGTCCGACACGGCGACCTC		
PRDX6	GGCCGCATCCGTTTCCACGA	280	NM_214408.1
	ACTGGATGGCAAGGTCCCGACT		
SOD1	TCCATGTCCATCAGTTTGGA	250	NM_001190422.1
	CTGCCCAAGTCATCTGGTTT		
SAA	GGCATCATTCCTCAAGGAAG	168	NM_001044552.1
	CTGATCACTTTAGCAGCCCA		
TNFα	CATGAGCACTGAGAGCATGA	170	NM_214022.1
	CGATAACTTCGAAGTGCAGT		
TXNRD1	CTTTACCTTATTGCCCGGGT	162	NM_214154.2
	GTTCACCGATTTTGTTGGCC		

**Table 3:** Average expression stability ranking of six candidate reference genes used in liver and duodenal mucosa tissue to their stability score M

	**Liver**		**Duodenum**	
**Ranking**	**Gene**	*M*-value*	**Gene**	***M*-value**
**Most stable**	ATP5G1	0.059	SDHA	0.066
	RPS9	0.057	GAPDH	0.070
	SDHA	0.062	GPI	0.077
	ACTB	0.068	ATP5G1	0.078
	GPI	0.074	ACTB	0.086
**Least stable**	GAPDH	0.110	RPS9	0.102

* calculated by the Microsoft Excel-based application GeNorm.

### DNA-binding activities of NF-κB and Nrf2 in duodenal mucosa

For the measurement of DNA-binding activities of NF-κB and Nrf2, 200 mg scraped mucosa cells were used to prepare nuclear extracts with a Nuclear Extract Kit (Active Motif, Rixensart, Belgium) according to the manufacturer’s protocol. Protein concentrations were determined by the bicinchoninic acid protein assay kit (Interchim, Montluçon, France) with bovine serum albumin (BSA) as standard. 20 μg (NFκB) and 30 μg (Nrf2) of nuclear protein were used to quantify NF-κB and Nrf2 transactivation with the transcription factor assays TransAM™ NFκB and TransAM™ Nrf2 (Active Motif, Rixensart, Belgium) according to the manufacturer’s protocol. Oligonucleotides containing the NF-κB consensus site (5^′^-GGGACTTTCC-3^′^) or an antioxidant response element (ARE, 5^′^GTCACAGTGACTCAGCAGAATCTG^′^3) for Nrf2 binding have been immobilised on a 96-well plate. The active forms of NF-κB and Nrf2, contained in the nuclear extracts, specifically bind to these oligonucleotides. 1.25 μg nuclear protein of Nrf2 transfected COS-7 cells or 2.5 μg nuclear protein of stimulated Jurkat cells (NF-κB translocation stimulated with 12-O-Tetradecanoylphorbol-13-acetate and calcium ionophore) were used as positive controls and nuclear extract from untransfected COS-7 cells and unstimulated Jurkat cells were used as negative controls.

Binding activity of the transcription factors to their consensus sequence was detected with specific primary antibodies against DNA bound NF-κB or Nrf2, respectively. Addition of a HRP-conjugated secondary antibody, followed by a developing solution and a stop solution, provides a colorimetric readout which was quantified by measurement of absorbance at 450 nm with a reference wavelength of 655 nm.

### High-performance thin-layer chromatography (HPTLC) analysis of malvidin 3-glucoside

Anthocyanes from GSGME and diets were extracted with acidic methanol (0.5% of hydrochloric acid in methanol). Anthocyanes from the extracts were separated on HPTLC plates silica gel 60 and a solvent mixture consisting of ethyl acetate/2-butanone/formic acid/water (7:3:1.2:0.8, v/v/v/v). Quantification of malvidin 3-glucoside was performed by external calibration and absorbance measurement at 530 nm using the TLC Scanner 4 (CAMAG, Muttenz, Switzerland). The concentration of malvidin 3-glucoside was determined in GSGME and in the diets. Analysis in the diets was performed in pooled samples of the diets administered in phases I and II.

### Cryosectioning for light microscopy

A duodenal tissue sample of each pig was removed and fixed in 4% paraformaldehyde (MERCK, Darmstadt, Germany) overnight at 4°C. Samples were washed three times for 10 min with 1xPBS followed by incubation in cryoprotectant 1x PBS solution containing 30% sucrose for 24 h. The fixed tissues were embedded in Tissue-Tek (Hartenstain, Wurzburg, Germany) and cryosectioned on a cryostat microtome (Microme HM 500, MICROM international GmbH, Walldorf, Germany) to 20 μm thickness. The sections were analyzed by light microscopy (Leica DMI 6000B) at 100× magnification for calculating the ratio of villus length to crypt depth, which were reported as mean length of 15 well oriented and representative villi and crypts from each pig.

### Statistical analysis

Means of the two groups were compared by Student’s *t*-test using the Minitab Statistical Software Rel. 13.0 (Minitab, State college, PA, USA). Means were considered significantly different for *P* < 0.05. Values in the text are means ± SD.

## Results

### Concentration of malvidin 3-glucoside in GSGME and diets

The concentration of malvidin 3-glucoside, the major anthocyanin in grapes, was 681 ± 45 μg/g (n = 3) in the GSGME. The concentration of malvidin 3-glucoside in the diets supplemented with 1% of GSGME (determined in a pooled sample of phase I and phase II diet) was 7.9 ± 0.4 μg/g (n = 3); in the control diet, malvidin 3-glucoside was not detectable (<0.1 μg/g).

### Growth performance of the pigs

There was no difference in final body weights, average daily gains, and daily feed intake between the two groups of pigs (Table 4). However, the gain:feed ratio was increased in the group fed the diet containing GSGME compared to the control group (*P* < 0.05; Table 4).

**Table 4:** Growth performance parameters of pigs fed a control diet or a diet supplemented with 1% GSGME

	**Control**	**GSGME**
Initial body weight (kg)	11.7 ± 0.4	11.5 ± 0.5
Final body weight (kg)	30.7 ± 2.1	31.9 ± 1.9
Daily feed intake (g)	1090 ± 100	1113 ± 82
Daily body weight gain (g)	681 ± 75	726 ± 62
Gain:feed ratio (g gain/kg feed)	624 ± 24	652 ± 29*

Results are shown as mean ± SD (n = 12/group). *Means are significantly different, *P* < 0.05.

### Concentrations of α-tocopherol, TBARS and antioxidative capacity

In order to assess the antioxidative status of the pigs, concentrations of α-tocopherol and TBARS (as a measure of lipid oxidation products) in plasma and liver as well as the plasma antioxidant capacity were determined. Concentrations of α-tocopherol and TBARS in plasma and in liver were not different between the two groups of pigs (Table 5). The antioxidative capacity of plasma was also not different between the two groups of pigs (Table 5).

**Table 5:** Concentrations of α-tocopherol, TBARS and antioxidative capacity in pigs fed a control diet or a diet supplemented with 1% GSGME

	**Control**	**GSGME**
**TBARS**		
Liver, nmol/g	26 ± 9	29 ± 9
Plasma, nmol/g	0.83 ± 0.27	0.95 ± 0.20
Plasma, mmol/mol lipid^#^	0.33 ± 0.06	0.34 ± 0.05
**α-tocopherol concentration**		
Liver, nmol/g liver	30 ± 10	28 ± 2
Plasma, μmol/L	5.7 ± 1.3	5.8 ± 1.3
Plasma, mmol/mol lipids^#^	1.9 ± 0.4	1.9 ± 0.3
**Antioxidative capacity**		
Plasma, mM Trolox equivalents	319 ± 31	307 ± 39

Results are shown as mean ± SD (n = 12/group). ^#^Sum of triglycerides and cholesterol.

### Activity of NF-κB and relative mRNA abundances of NF-κB target genes in duodenal mucosa

**Figure 1:**
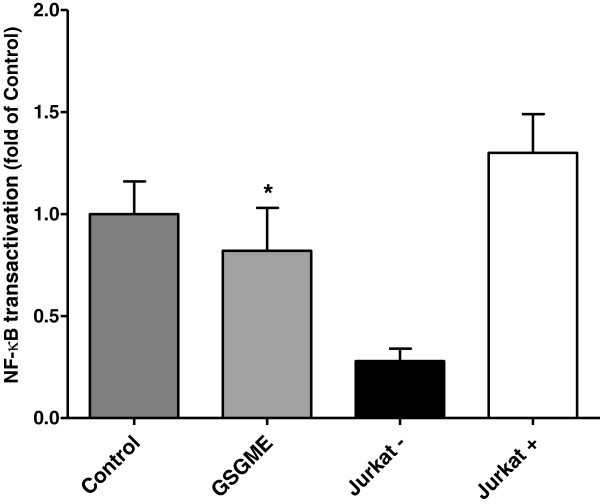
**Relative DNA-binding activity of NF-κB in the nuclear extract of duodenal mucosa of pigs fed the control diet or GSGME containing diet.** Nuclear extract from Jurkat cells without stimulation was used as negative control (Jurkat -) and nuclear extract from Jurkat cells with NF-κB stimulation were used as positive control (Jurkat +). Bars represent mean ± SD of 12 pigs per group and are expressed as fold of NF-κB DNA-binding activity of the control group. *Significantly different from the control group; *P* < 0.05.

**Figure 2:**
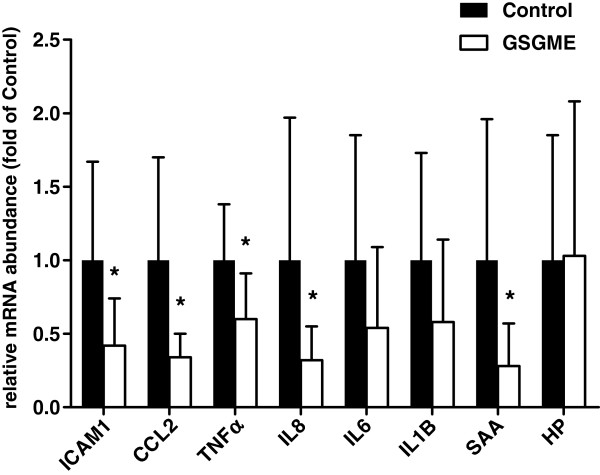
**Relative mRNA abundance of NF-κB target genes ICAM1, CCL2, TNFα, IL8, IL6, IL1B, SAA and HP in the duodenal mucosa of pigs fed the control diet or GSGME containing diet.** Bars represent mean ± SD of 12 pigs per group and are expressed as fold of relative mRNA abundance of the control group. *Significantly different from the control group; *P* < 0.05.

DNA-binding activity of NF-κB in the duodenal mucosa of the pigs was significantly decreased in the GSGME group compared to the control group (*P* < 0.05; Figure 1). In agreement with a reduced transactivation of NF-κB, pigs fed the diet containing GSGME had lower concentrations of relative abundances of several NF-κB target genes involved in inflammation [intercellular adhesion molecule 1 (ICAM1), chemokine (C-C motif) ligand 2 (CCL2), tumor necrosis factor α (TNFα), interleukin 8 (IL8)] and acute phase response [serum amyloid A (SAA)] in duodenal mucosa than control pigs (*P* < 0.05, Figure 2). In contrast, relative mRNA abundances of interleukin 6 (IL6), interleukin 1β (IL1B) and haptoglobin (HP), which are also target genes of NF-κB, were not significantly different between both groups (Figure 2).

### Activity of Nrf2 and relative mRNA abundances of Nrf2 target genes in duodenal mucosa

**Figure 3:**
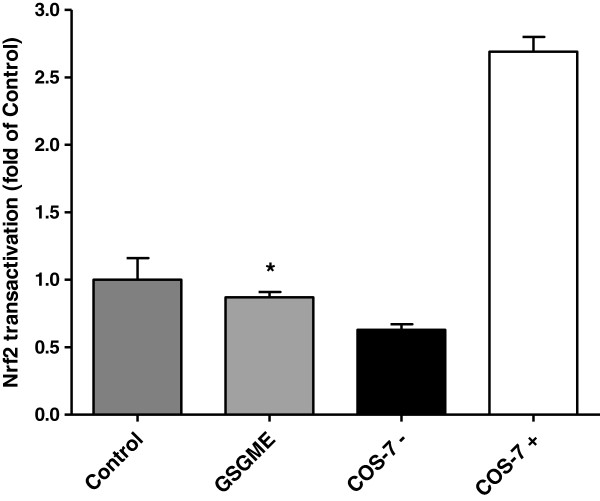
**Relative DNA-binding activity of Nrf2 in the nuclear extract of duodenal mucosa of pigs fed the control diet or GSGME containing diet.** Nuclear extract from unstimulated COS-7 cells was used as negative control (COS-7 -) and nuclear extract from Nrf2 transfected COS-7 cells was used as positive control (COS-7 +). Bars represent mean ± SD of 12 pigs per group and are expressed as fold of Nrf2 DNA-binding activity of the control group. *Significantly different from the control group; *P* < 0.05.

**Figure 4:**
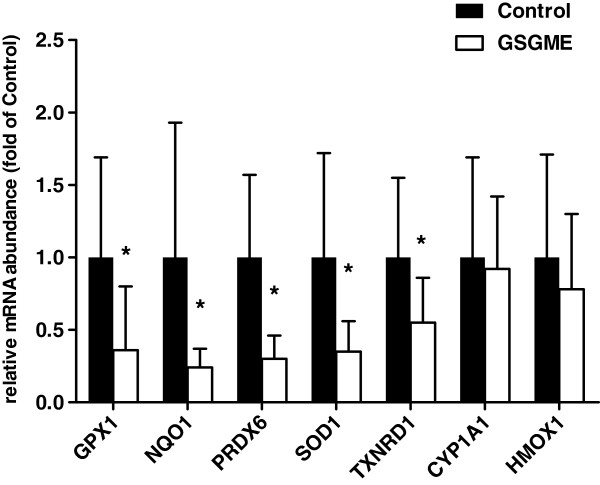
**Relative mRNA abundance of Nrf2 target genes GPX1, NQO1, PRDX6, SOD1, TXNRD1, CYP1A1 and HMOX1 in the duodenal mucosa of pigs fed the control diet or GSGME containing diet.** Bars represent mean ± SD of 12 pigs per group and are expressed as fold of relative mRNA abundance of the control group. *Significantly different from the control group; *P* < 0.05.

DNA-binding activity of Nrf2 in the duodenal mucosa of the pigs was significantly decreased in the GSGME group compared to the control group (*P* < 0.05; Figure 3). In agreement with a reduced transactivation of Nrf2, relative mRNA abundances of the Nrf2 target genes glutathione peroxidase 1 (GPX1), NAD(P)H dehydrogenase, quinone 1 (NQO1), peroxiredoxin 6 (PRDX6), superoxide dismutase 1 (SOD1) and thioredoxin reductase 1 (TXNR1), encoding proteins with antioxidant or cytoprotective functions, in the duodenal mucosa were lower in pigs fed the diet containing GSGME than in control pigs (P < 0.05, Figure 4). In contrast, relative mRNA abundances of cytochrome P450, family 1, subfamily A, polypeptide 1 (CYP1A1) and heme oxygenase 1 (HMOX1), two other Nrf2 target genes considered, in small intestinal mucosa, did not differ between both groups (Figure 4).

### Duodenal morphology

Pigs fed the diet containing GSGME had a significantly increased villus height:crypt depth ratio in the duodenum compared to pigs fed the control diet (2.11 ± 0.11 versus 1.94 ± 0.22; *P <* 0.05).

## Discussion

The major aim of this study was to assess the hypothesis that plant extracts rich in polyphenols are able to inhibit the inflammatory process in the duodenum of pigs. For this purpose, we used GSGME, a by-product of wine/grape juice processing, as a plant extract rich in flavonoids. The most abundant polyphenols in grape seeds (GS) are gallic acid, catechin, epigallocatechin-3-gallate, epigallocatechin, epicatechin-3-gallate, epicatechin, and proanthocyanidins [25]. Grape marc (GM) contains less procyanidins (with the exception of the procyanidin dimer B1) but contains significant amounts of anthocyanins, such as cyanidine 3-glucoside, malividin 3-glucoside, cyaniding and peonidin, which are absent in GS [25]. For technical reasons, we were not able to characterise the polyphenol spectrum in the product used. According to the supplier, the total polyphenol content of the GSGME product used was about 8.5%. The high content of polyphenols in this product was confirmed by HPTLC-determination of malvidin 3-glucoside, the quantitative most important pigment in red grape, whose mean concentration was 681 μg/g.

In accordance with the hypothesis underlying this study, we observed that feeding the polyphenol-rich GSGME reduces transactivation of NF-κB in the duodenal mucosa and in turn lowers transcript levels of various NF-κB target genes involved in the inflammatory process. These findings implicate that GSGME exerts anti-inflammatory effects in the duodenum of pigs. Although the study does not directly provide evidence for this, it is likely that the anti-inflammatory effect of the plant extract might have been induced by its high content of polyphenols. Thus, this study suggests that feeding of plants rich in polyphenols such as GSGME could provide a useful dietary strategy to suppress the inflammation process in the small intestine frequently occurring in pigs, particularly after weaning. The finding that polyphenols are able to suppress the inflammation process in duodenum of pigs agrees with several *in vitro*-studies using intestinal epithelial cells and *in vivo*-studies which were mainly performed in rodent models of acute or chronic colitis [26]. While most of these studies have been performed with green tea polyphenols, there are also some studies which showed inhibitory effects on the inflammation process of anthocyanins from grapes or GS in either intestinal epithelial cells [23,27] or intestine of rats with ulcerative colitis [28]. Based on these findings, supplementation of polyphenols has been even proposed as a complementary medicinal approach for treatment of inflammatory bowel disease [29]. Notably, in the present study, we did not use pigs with an experimentally induced intestinal inflammation due to ethical reasons. Based on the findings in rodent models of acute or chronic intestinal inflammation, we assume that GSGME might exert anti-inflammatory effects also in pigs with an acute intestinal inflammation process.

There are several studies showing that polyphenols have the potential to activate Nrf2 and in turn to enhance the expression of several antioxidative and cytoprotective genes in the small intestine [30,31]. Surprisingly, in the present study administration of the polyphenol rich GSGME did not increase but even reduced transactivation of Nrf2 and gene expression of several Nrf2 target genes in duodenum. Based on the fact that Nrf2 is activated by either ROS or by pro-inflammatory cytokines [32,33], we assume that the inhibition of Nrf2 signalling in the small intestine of pigs administered GSGME was simply due to its strong anti-oxidative and anti-inflammatory properties which might have suppressed the local production of ROS and pro-inflammatory cytokines in the surrounding of intestinal cells.

Several studies have shown that diverse polyphenols, particularly those present in green tea, are able to inhibit NF-κB and to activate Nrf2 in the liver [10,34]. Furthermore, studies exist dealing with the effects of polyphenols from GS or GM on the antioxidant system in the liver, mostly in rats or rabbits [35-37]. However, to our knowledge, less information is available about effects of polyphenols from GS or GM on the signalling pathways in the liver. In order to find out whether feeding GSGME could also have protective effects on the liver, we determined relative mRNA abundances of NF-κB and Nrf2 target genes in the liver of the pigs. However, no alterations of mRNA abundances of these genes were observed, indicating that feeding GSGME had no effect on NF-κB and Nrf2 signalling in the liver. A low intestinal bioavailability might be one possible reason for the lack of effect in this respect. Although there is only limited information available in the literature regarding the bioavailability of polyphenolic compounds, it has been suggested that the bioavailability of polyphenols might be in the range between 10 and 50%, depending on their chemical structure, the dose applied, the form of application and the species studied [38]. As the absorption of polymeric proanthocyanidins is negligible [39,40], the bioavailability of total polyphenols of GSGME might be comparably low.

Polyphenols have a great antioxidative potential. Therefore, it has been suggested that feeding diets rich in polyphenols could improve the antioxidative status of plasma and tissues and increase tocopherol concentrations due to a vitamin E-sparing effect [41]. However, the published literature regarding the effects of dietary polyphenols on tocopherol concentrations and the antioxidative status in plasma and tissues is inconclusive. While some studies in rats reported an increase of plasma and tissue tocopherol concentrations by feeding various types of flavonoids [42-44], other studies observed no effects [45-47]. In pigs, a tocopherol-sparing effect of quercetin has been observed under condition of a low dietary vitamin E concentration [48]. In contrast, in pigs fed diets with nutritionally adequate vitamin E concentrations, no increases of plasma and tissue tocopherol concentrations by supplementing dietary flavonoids were observed [49,50]. The study of Wiegand et al. [50] moreover shows that dietary flavonoids do not alter the expression of hepatic genes involved in transfer of tocopherols into plasma lipoproteins, decomposition and excretion into the bile, meaning that metabolism of vitamin E remained unchanged. The present study confirms that dietary polyphenols from GS and GM do not improve the vitamin E status, and do not improve the antioxidant status of pigs with an adequate supply of dietary vitamin E (of around 100 mg α-tocopherol acetate/kg diet).

In this study, we found that administration of GSGME improves the gain:feed ratio in growing pigs suggesting that either digestibility of nutrients from the diets or intermediary utilisation of nutrients was improved. To our knowledge, there are no other comparable studies in pigs available in the literature dealing with the effects of moderate amounts of dietary grape by-products on the growth of pigs. There are, however, a few studies in broiler chicks showing beneficial effects of polyphenol-rich grape products on digestibility of nutrients and feed efficiency. In the study of Viveros et al. [51], feeding polyphenol-rich grape pomace extract (60 g/kg diet) improved the gain:feed ratio in broilers at 21 d of age. In that study, also an increase of the villus height:crypt depth ratio at the jejunum and a shift in the ileal bacterial populations (increase in beneficial bacteria such as Enterococcus, decrease in potential pathogens such as Clostridium) was observed in broilers fed the grape pomace extract. In the study of Brenes et al. [52], feeding diets containing 0.6 to 3.6 g GS extract did not improve the gain:feed ratio but improved ileal protein digestibility in 21 d-old broilers. In the study of Wang et al. [53], administration of a GS proanthocanidin extract lowered mortality and increased growth in broilers infected with *E. tenella*. Weaning in pigs is associated with a strong decrease in the villus height:crypt depth ratio in the small intestine which in turn leads to a reduced digestive capacity [54]. Interestingly, the present study is in agreement with the broiler study of Viveros et al. [51] in observing an increased villus height:crypt depth ratio in the small intestine by feeding a polyphenol rich GS by-product. It is assumed that an increased villus height leads to an improvement of digestive and absorptive function of the intestine as a result of increased absorptive surface, expression of brush border enzymes and nutrient transport systems [54,55]. Thus, although we did not determine the digestibility of nutrients in this study, it is possible that the increase in the gain:feed ratio of pigs administered GSGME was due to an improvement of the digestibility of nutrients. Based on the observations in broilers [51], the effects on the microflora could contribute to the beneficial effects of GSGME.

## Conclusions

In conclusion, the present study shows that oral administration of a polyphenol rich GSGME suppresses the activity of NF-κB in small intestine of pigs and thus might provide a useful dietary strategy to inhibit inflammation in the gut frequently occurring in pigs, particularly after weaning. Feeding GSGME did not influence vitamin E status and the antioxidant system of the pigs but improved the gain:feed ratio in the pigs. In overall, the study suggests that polyphenol-rich plant extracts such as GSGME could be useful feed supplements in pig nutrition, in order to improve both animal health and performance.

## Abbreviations

ARE: Antioxidant response element; CCL2: Chemokine (C-C motif) ligand 2; CYP1A1: Cytochrome P450, family 1, subfamily A, polypeptide 1; GPX1: Glutathione peroxidase 1; GSGME: Grape seed and grape marc meal extract; HMOX1: Heme oxygenase 1; HP: Haptoglobin; HPTLC: High-performance thin layer chromatography; ICAM1: Intercellular adhesion molecule 1; IL: Interleukin; Keap-1: Kelch-like ECH-associated protein 1; Nrf2: Nuclear factor erythroid 2-related factor 2; NF-κB: Nuclear factor kappa B; NQO1: NAD(P)H dehydrogenase, quinone 1; PRDX6: Peroxiredoxin 6; ROS: Reactive oxygen species; SAA: Serum amyloid A; SOD1: Superoxide dismutase 1; TEAC: Trolox equivalent antioxidant capacity; TBARS: Thiobarbituric acid-reactive substances; TXNRD1: Thioredoxin reductase 1.

## Competing interests

The authors declare that they have no competing interests.

## Authors’ contributions

DKG participated in the study design and development of hypotheses, acquisition of the data, statistical analyses, interpretation of results and writing of the manuscript. AF, EM, JD, GW and RR performed laboratory analyses and statistics. KE participated in the study design and development of hypotheses, interpretation of results, and writing the manuscript. All authors have read and approved the final manuscript.
